# Life after COVID-19: R WE going to help?

**DOI:** 10.2217/cer-2020-0056

**Published:** 2020-05-11

**Authors:** Sreeram V Ramagopalan, Radek Wasiak

**Affiliations:** ^1^Global Access, F. Hoffmann-La Roche Ltd, Grenzacherstrasse 124, CH-4070, Basel, Switzerland; ^2^Cytel Ltd, Hamilton House, Mabledon Place, London WC1H 9BB, UK

**Keywords:** coronavirus, COVID-19, real-world evidence

The threat of COVID-19 has led to an unprecedented level of cooperation among academia, industry, policy makers and public health officials to find ways to diagnose, prevent and cure the condition. Although key work is interventional trials testing vaccines and treatments, observational studies (or real-world evidence [RWE]) have proved valuable in aiding decision-making [1–4]. Awareness of how RWE is generated, its limitations and the biases presented that need to be addressed using statistical methods to gain meaningful insights, is now commonplace and should not impede its use and acceptance [5]. The research activities related to COVID-19 should hopefully help end the often unproductive debate about the superiority of randomized clinical trials over RWE [6].

Analogizing to the 2008 global financial crisis, which negatively impacted individual health and access to care and forced changes to service provision in budget constrained healthcare systems [7,8], we will likely observe material changes after the pandemic is over. What is different about this shock to our lives and economies is the substantially greater existence of data and the ability to process this in vast quantities.

Data-enabled decision-making has the potential to offer significant benefits to patients, health systems and society as a whole; however, in order to fully capitalize on this, more data need to be made available. Imagine if full medical records were available on people worldwide, in real time, potentially also linked to their social media and wearable technology accounts. If used for research purposes (complying with all privacy and legal guidance) this could have meaningfully assisted efforts to build predictive models for COVID-19 and associated complications [9] and to aid flattening of the COVID-19 transmission curve, translating to extensive global health and economic gains.

How could more data help after the COVID-19 pandemic? Cancer is a highly relevant example where there are high unmet needs and limited data available. A survey of over 4000 cancer patients in more than ten countries by All. Can [10], found that there were often delays in disease diagnosis, inefficiencies in cancer management and that the disease caused considerable financial challenges for sufferers through loss of earnings. It is likely that these findings can be extrapolated to some extent to other diseases. Less fragmented data can help in all aspects of the patient journey, with the ultimate goal of improving patient (and caregiver) holistic well-being (physically, mentally and financially) and concomitantly reducing burden and cost to healthcare systems.

New methods can help convert data to insight; artificial intelligence techniques have already been shown to assist on cancer image data to improve diagnosis [11]. With prospective validation of these techniques with imaging data directly from the clinic, deployment of these systems could reduce strain on the healthcare workforce and ensure high-risk patients are rapidly followed up. Although precision medicine is currently most applicable to oncology, where genomically targeted therapy can lead to better clinical outcomes [12], medicine can become more personalized and more efficient with more complete and less fragmented data. Actionable information such as quality of life data are important and whether this is tracked using wearables, social media or via self-monitoring apps [13,14], it can empower both patients and healthcare practitioners to address any deterioration in symptoms that otherwise may escape detection in between routine clinical visits. Improvement of patient outcomes potentially not only enables them (and their caregivers) to carry on working, which has a positive societal impact, it also lessens healthcare resource use.

The need to develop new medicines remains, despite most attention being placed on healthcare interventions to support ending the COVID-19 pandemic. Real world data can provide supportive information to help with designing (e.g., defining appropriate [potentially digital and more patient-centric] end points to use) and interpreting (e.g., understanding generalizability of trial populations) randomized clinical trials. Real world data can also be used to act as a comparator when it is difficult or unethical to run a placebo-controlled study. All of these efforts can assist in bringing patients faster access to medicines and ultimately improving their lives and bettering society in general. While the health and financial advantages of more data availability are tangible, there are nevertheless data privacy, quality, fragmentation, infrastructure and logistical challenges that need to be surmounted [15–17]. However, COVID-19 has shown that when a crisis hits, we all can work together and commit to a greater good. Perhaps this is an opportunity that the pandemic is affording us: putting things in place now to improve health and wealth outcomes for all and limit future healthcare emergencies.
